# The association between vitamin D status and clinical events in high-risk older patients with non-ST elevation acute coronary syndrome undergoing invasive management

**DOI:** 10.1371/journal.pone.0217476

**Published:** 2019-06-12

**Authors:** Benjamin Beska, Danny Chan, Sophie Gu, Weiliang Qiu, Helen Mossop, Dermot Neely, Vijay Kunadian

**Affiliations:** 1 Institute of Cellular Medicine, Faculty of Medical Sciences, Newcastle University, Newcastle upon Tyne, United Kingdom; 2 Cardiothoracic Centre, Freeman Hospital, Newcastle upon Tyne Hospitals NHS Foundation Trust, Newcastle upon Tyne, United Kingdom; 3 Channing Division of Network Medicine, Department of Medicine, Brigham and Women's Hospital/Harvard Medical School, Boston, MA, United States of America; 4 Institute of Health and Society, Newcastle University, Newcastle upon Tyne, United Kingdom; 5 Department of Biochemistry, Newcastle upon Tyne Hospitals NHS Foundation Trust, Newcastle upon Tyne, United Kingdom; Azienda Ospedaliero Universitaria Careggi, ITALY

## Abstract

There is a higher incidence of vitamin D deficiency in older adults. This may play a plausible mechanistic role in the occurrence of increased adverse events after non-ST elevation acute coronary syndrome (NSTEACS). This study investigated whether total vitamin D levels at the time of presentation predicted adverse outcomes in older adults undergoing invasive management of NSTEACS. Of the 629 patients screened, 300 high-risk older adults with NSTEACS managed by an invasive strategy were recruited. Serum total 25-hydroxyvitamin D was measured at index presentation. The primary outcome was defined as 1-year composite of all-cause mortality, acute coronary syndrome (ACS), unplanned repeat revascularisation, significant bleeding or stroke. Mean age was 80.5±4.8 years (61.9% male). Median vitamin D level was 29.5nmol/L [interquartile range IQR 16.0–53.0 nmol/L] and was split equally by the median for analysis forming two groups: high (median vitamin D 53.0 nmol/L [IQR 40.0–75.0]) and low (16.0 nmol/L [11.0–23.0]). The primary outcome occurred in 76 patients (25.9%); 32 (21.9%) in the low group and 44 (29.9%) in the high group, p = 0.12. Multivariable analyses showed no significant difference in the primary composite outcome at 1 year between the low and high group of baseline serum vitamin D (Hazard Ratio 1.20 [95% Confidence Interval 0.72–2.0], p = 0.48). Serum total vitamin D, measured at the time of angiography, was not associated with adverse outcomes at one year in this high-risk older cohort of patients with NSTEACS undergoing invasive management.

## Introduction

Older patients have a higher incidence of adverse outcomes after acute coronary syndrome (ACS), with a 15.7 increased odds of in-hospital mortality in patients ≥85 years old compared to those <45 years old.[1, 2] Due to under-representation of this older cohort in studies of ACS, it is not clear why this population remains at increased risk.[3–5] The ageing population is likely to further increase the proportion of older patients presenting with non-ST elevation acute coronary syndrome (NSTEACS), the predominant ACS phenotype in this age group.[6] Studies involving age-appropriate biomarkers therefore are required to help understand this inequality in outcomes. Vitamin D is a hormone obtained largely through the action of ultraviolet B light on the cutaneous tissues, with sequential hydroxylation of activated skin pre-cursors by liver and kidney producing biologically active 1,25-dihydroxyvitamin D.[7] Although associated with the traditional target tissues of kidney, gut and bone and involved in inorganic ion metabolism, there is increasing evidence that low serum levels of vitamin D may be an important biomarker of an increased risk of cardiovascular disease (CVD).[7]

There is established evidence of a relationship on an epidemiological scale, with a number of large meta-analyses linking lower serum vitamin D with both incidence of CVD and an increased CVD mortality in largely population-based studies.[8, 9] This is clinically important, given that more than one billion people worldwide are vitamin D non-sufficient,[10] with incidence increasing up to 80% in older adults.[11] Previous studies have investigated the prognostic value of vitamin D in ACS,[12–14] however it is not conclusively known whether levels of total vitamin D at the time of index presentation influence the incidence of adverse outcomes in NSTEACS older patients undergoing invasive management. We aimed to prospectively investigate this relationship in high-risk older adults presenting with NSTEACS treated with an invasive strategy.

## Methods

Older patients aged ≥65 years referred to either the Freeman Hospital, Newcastle upon Tyne (Newcastle upon Tyne Hospitals NHS Foundation Trust) or James Cook University Hospital, Middlesbrough (South Tees Hospitals NHS Foundation Trust) for invasive management of NSTEACS were recruited into the ICON1 (**I**mprove **C**ardiovascular **O**utcomes in High Risk Patie**N**ts, Clinicaltrials.gov ID: NCT01933581) study between November 2012 and December 2015. After recruitment commencement, we focussed our recruitment on high risk older patients aged 75 and over. The ICON1 study is a multi-centre prospective cohort study that aims to determine the predictors of adverse outcomes following interventional management of NSTEACS in older patients, with an overall aim of producing a quantitative risk score for this population, the FRAIL-HEART score.[15] [16, 17]

The full study protocol has been previously published.[18] This study has been carried out in accordance with the Declaration of Helsinki. Ethical approval was gained from National Research Ethics Service (12/NE/0160). Written, informed consent was taken from all participants prior to enrolment into the study. Baseline assessment occurred during the index admission when the patient presented for invasive management of NSTEACS. Frailty using the Rockwood score and using the Fried Frailty Index, derived from the Cardiovascular Health Study with a score of 0 is categorised as robust, 1–2 as pre-frail and ≥3 as frail.[19] Cardiovascular status was assessed with New York Heart Association (NYHA) grade. Cognitive status was assessed using the Montreal Cognitive Assessment (MOCA).[20] Patient-reported health-related quality of life was assessed using the Short Form 36 (SF-36) surveys, reported as norm-based scores.[21] Charlson co-morbidity index[22] and Global Risk of Acute Coronary Event (GRACE) 2.0 score were also calculated.

Assessment of non-invasive cardiovascular parameters was performed at baseline. Endothelial function was measured with peripheral artery tonometry (EndoPAT 2000 device, ITAMAR Medical) with previously described methodology,[23] reported as natural log reactive hyperaemia index (lnRHI). Vascular stiffness is assessed through evaluation of carotid-femoral pulse-wave velocity (PWV) with the Vicorder device (SMT Medical).

Blood was drawn from patients immediately prior to angiography. Serum 25-hydroxyvitamin D was analysed using an automated direct competitive chemiluminescent immunoassay. Three separate assay platforms were used during the period of recruitment: DiaSorin Liaison (November 2012–July 2013), Roche Diagnostics Modular E170 (July 2013–May 2015) and Roche Cobas 602 (May 2015–December 2015). For more information on the assay platforms see **S1 File**.

The *a priori* primary composite outcome was defined as incidence of all-cause death, acute coronary syndrome, unplanned repeat revascularisation, stroke and major bleeding (Bleeding Academic Research Consortium-defined type 2 or greater) at 1 year. Detailed definitions of study outcome measures have been previously described.[18] Patients were followed up in the study outpatient clinics at 1 year. If patients were unable to attend follow-ups visits, they were followed up by phone call or through GP records.

Statistical analysis was performed using SPSS version 24 (IBM Corporation). Statistical significance was defined as two-tailed *P* ≤ 0.05. Those variables classed as normally distributed were reported as mean ± standard deviation (SD). Variables that were non-normally distributed were reported as median [interquartile range (IQR)]. Vitamin D was a non-normally distributed variable and was split into two equal groups via the median for analysis. Baseline differences in patient characteristics were assessed between groups of vitamin D with one-way ANOVA for normally distributed continuous data, Kruskal-Wallis testing for non-normally distributed continuous data, and chi-squared test (χ2) or Fisher exact test (as appropriate with expected cell counts of <5) for categorical variables.

Kaplan-Meier survival analysis was performed to analyse the differences in cumulative event-free survival between groups of serum vitamin D, tested for statistical significance with the Log-rank test. Cox proportional hazards regression was used to model the risk of incidence of the primary composite end-point. Three models were constructed. Model 1 was an unadjusted univariate analysis. Model 2 was adjusted for age, sex and month of blood collection. Model 3 was a multivariate analysis with hierarchical addition of covariates which differed between groups at baseline. Model 4 was additionally adjusted for the use of vitamin D supplementation, producing a fully-adjusted model. Internal validation of the fully adjusted model (Model 4) was performed through bootstrapping the original data set by sampling 1000 replacement bootstrap samples.

Ordinal regression was used to model the association between baseline variables of interest and groups of serum baseline vitamin D. This was adjusted for through multivariate analysis with hierarchical addition of covariates which differed between groups at baseline.

The Hsieh and Lavori method was used to calculate the statistical power for incidence of the primary composite outcome, using a type I error rate of 0.05, with the outcome rate estimated conservatively at 5%. A sample size of 300 participants provided 80% power to detect an association between the covariates and the composite primary outcome, with a hazard ratio of >2.0.[18]

## Results and discussion

**Fig 1:**
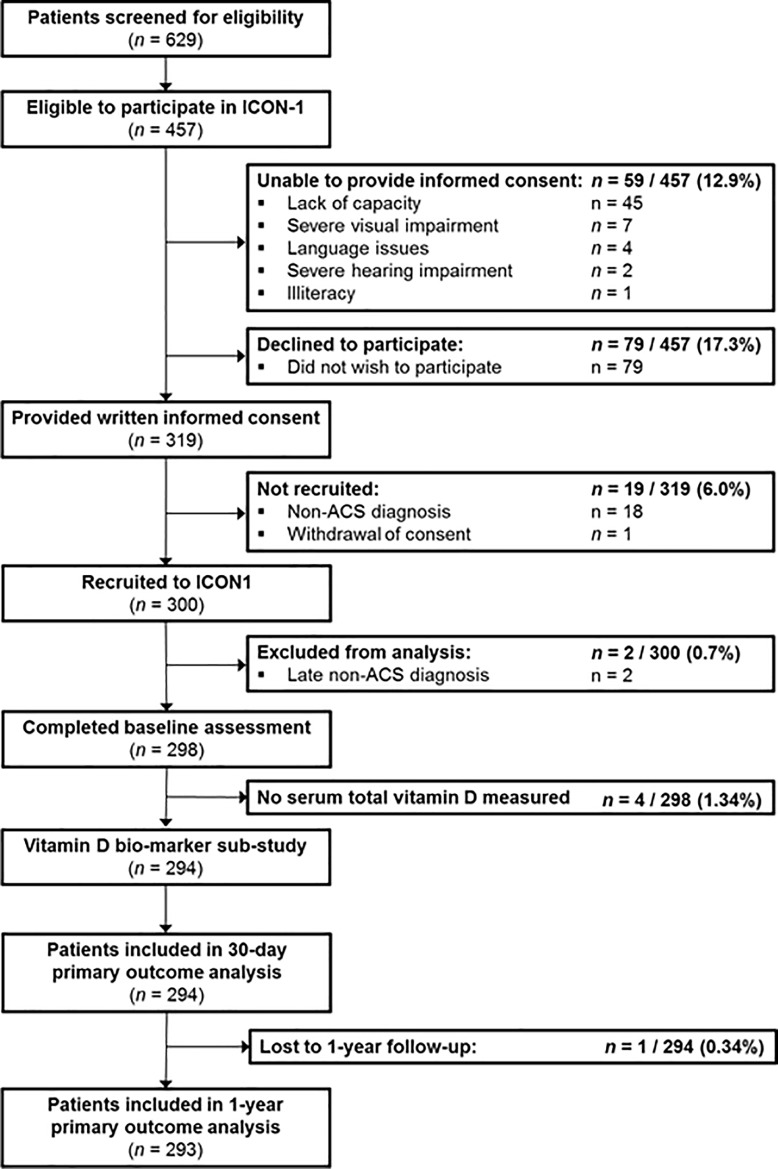
Flow diagram for the ICON-1 vitamin D biomarker sub-study.

A flow diagram of recruitment to the ICON1 trial is detailed in **Fig 1**. 629 patients were screened. Two patients out of the 300 recruited had a non-ACS diagnosis following angiography and were excluded. Of the remaining 298 patients, 294 (98.7%) had serum total vitamin D measured. One patient was lost to 1 year follow-up, meaning the total sample available for analysis at 1 year was 293.

The median serum vitamin D of the cohort was 29.5 nmol L^-1^ [interquartile range IQR 16.0–53.0]. A split by the median created two equal groups for analysis: High serum vitamin D (median 53.0 nmol L^-1^ [40.0–75.0], n = 147) and low serum vitamin D (median 16.0 nmol L^-1^ [11.0–23.0], n = 147). Baseline characteristics are summarised in **Table 1**, grouped by vitamin D level. The median age of the cohort was 80.5 years [IQR 77.2–83.5] and 61.9% were male. Overall, 241 patients (82.0%) presented with non-ST elevation myocardial infarction (NSTEMI) and 53 patients (18.0%) presented with unstable angina. All patients underwent coronary angiography, and subsequently 244 (83.0%) were managed with percutaneous coronary intervention (PCI), 10 (3.4%) were managed with coronary artery bypass grafting and 40 (13.6%) were managed conservatively with medication alone.

**Table 1:** Baseline characteristics of study participants grouped by serum vitamin D.

		Baseline serum vitamin D		
	Total (N = 294)	High ≥29.5 nmol L^-1^ N = 147	Low <29.5 nmol L^-1^ N = 147	*P value*
**Demographic details**				
Age (years)	80.5 (±4.8)	79.9 (±4.7)	81.0 (±4.9)	**0.04**
Male, n (%)	182 (61.9)	99 (67.3)	83 (56.4)	0.06
Clinical characteristics*				
Heart rate on admission (bpm)	74.6 (±18.0)	74.9 (±19.3)	74.3 (±16.6)	0.80
Systolic blood pressure (mmHg)	144.3 (±25.3)	143.7 (±25.4)	144.9 (±25.2)	0.70
Body mass index (kg m^-2^)	27.2 (±4.6)	26.8 (±4.4)	27.6 (±4.8)	0.13
GRACE 2.0 score	130.2 (±19.8)	128.2 (±18.7)	132.2 (±20.7)	0.10
NYHA class III or IV, n (%)	55 (18.8)	25 (17.1)	30 (20.4)	0.52
**Presentation and management strategy**, n (%)				
NSTEMI	241 (82.0)	123 (83.7)	118 (80.3)	0.58
UA	53 (18.0)	24 (16.4)	29 (19.7)	0.58
PCI	244 (83.0)	125 (85.0)	119 (81.0)	0.35
CABG	10 (3.4)	4 (2.7)	6 (4.1)	0.52
Medication alone	40 (13.6)	18 (12.2)	22 (15.0)	0.46
**Past medical history**, n (%)				
Hypertension	217 (73.8)	99 (67.3)	118 (80.3)	**0.01**
Diabetes	71 (24.1)	35 (23.8)	36 (24.5)	0.89
Smoking status Current smoker Ex-smoker Never smoker	21 (7.1) 146 (49.7) 124 (42.2)	9 (6.1) 70 (47.6) 65 (44.2)	12 (8.2) 76 (51.7) 59 (40.1)	0.52
High cholesterol	172 (58.5)	87 (59.2)	85 (57.8)	0.06
Family history of IHD	92 (31.5)	48 (32.9)	44 (30.1)	0.25
Renal impairment	58 (19.7)	23 (15.6)	35 (23.8)	0.08
Previous MI	97 (33.0)	33 (22.4)	64 (43.5)	**0.0001**
Previous angina	122 (41.5)	54 (36.7)	68 (46.3)	0.10
Previous PCI	59 (20.1)	25 (17.0)	34 (23.1)	0.19
Previous CABG	16 (5.4)	7 (4.8)	9 (6.1)	0.61
Previous TIA/stroke	48 (16.3)	20 (13.6)	28 (19.0)	0.21
Atrial fibrillation	42 (14.3)	21 (14.3)	21 (14.3)	1.0
Peripheral vascular disease	29 (9.9)	18 (12.2)	11 (7.5)	0.17
COPD	52 (17.7)	22 (15.0)	30 (20.4)	0.22
Malignancy	28 (9.5)	15 (10.2)	13 (8.8)	0.69
CCF	24 (8.2)	5 (3.4)	19 (12.9)	**0.003**
**Frailty, co-morbidity and quality of life**				
Charlson co-morbidity index (points)	5.25 (±1.81)	4.95 (±1.65)	5.54 (±1.91)	**0.03**
MOCA (points)	25.2 (±3.2)	25.3 (±3.3)	25.0 (±3.1)	0.63
SF-36, PCS (points)	36.0 (±11.7)	36.0 (±11.5)	36.1 (±11.9)	0.65
SF-36, MCS (points)	50.7 (±9.8)	50.7 (±9.6)	50.6 (±10.0)	0.79
Fried Frailty Index n (%) Robust Pre-frail Frail	56 (19.1) 157 (53.6) 80 (27.3)	35 (24) 78 (53.4) 33 (22.6)	21 (14.3) 79 (53.7) 47 (32.0)	0.05
Rockwood Score n (%) Score 1–2 Score 3–4 Score 5–7	93 (31.7) 165 (56.3) 35 (11.9)	60 (41.1) 72 (49.3) 14 (9.6)	33 (22.4) 93 (63.3) 21 (14.3)	**0.003**
**Vitamin D supplementation**				
At baseline n (%)	34 (11.6)	24 (16.3)	10 (6.8)	**0.011**
At 1 year follow-up^**†**^ n (%)	54 (19.4)	34 (23.1)	20 (13.6)	**0.02**
Biochemical parameters*				
Haemoglobin (g L^−1^)	131.4 (±1.90)	134.1 (±1.93)	128.6 (±1.82)	**0.01**
White cell count (10^9^ L^−1^)	7.9 (6.6–9.7)	7.8 (6.5–9.4)	8.1 (6.9–10)	0.13
Total cholesterol (mmol L^−1^)	4 (3.3–4.9)	4.2 (3.4–5)	4 (3.2–4.9)	0.24
Creatinine (μmol L^−1^)	101.4 (±33.4)	98.8 (±28.0)	104.1 (±38.1)	0.17
eGFR (mLmin^−1^ 1.73m^−2^)	51.6 (41.9–65.4)	54.4 (43.7–67.9)	50.9 (40.9–64.8)	0.15
Peak troponin (ng L^−1^)	120.5 (40–417)	126 (42–512)	116 (36–352)	0.30
hsCRP (mg L^−1^)	4 (1.3–9.4)	3.5 (1.1–7)	4.5 (1.7–12)	**0.04**
Total vitamin D (nmol L^−1^)	29.5 (16–53)	53 (40–75)	16 (11–23)	**0.0001**
Non-invasive cardiovascular parameters*				
Vascular stiffness, PWV (m s^−1)^	9.32 (±2.0)	9.21 (±2.1)	9.42 (±2.0)	0.06
Endothelial function, lnRHI	0.56 (0.42–0.73)	0.58 (0.44–0.76)	0.55(0.44–0.69)	0.17

*Normally distributed continuous variables are reported as mean (±SD), non-normally distributed continuous variables are reported as median [IQR]. Statistically significant P ≤ 0.05) results are displayed **bold**. † Vitamin D supplementation status was collected in 278 patients at follow-up.

BPM, beats per minute; CABG, coronary artery bypass graft; CCF, chronic cardiac failure; COPD, chronic obstructive pulmonary disease; eGFR, estimated glomerular filtration rate; GRACE, Global Registry of Acute Coronary Events; hsCRP, high sensitivity C-reactive protein; IHD, ischaemic heart disease; lnRHI, natural log reactive hyperaemia index; MCS, mental component score; MI, myocardial infarction; NSTEMI, non-ST segment elevation myocardial infarction; NYHA, New York Heart Association; PCI, percutaneous coronary intervention; PCS, physical component score; PWV, pulse wave velocity; SD, standard deviation; SF-36, Short Form survey; TIA, transient ischaemic attack and UA, unstable angina.

There was no difference in management decision between baseline serum vitamin D levels ≥29.5 nmol L^-1^ (high group) vs. <29.5 nmol L^-1^ (low group) (*P* > 0.05). There was a significant difference between groups in age (*P* = 0.044), history of hypertension (*P* = 0.012), previous myocardial infarction (MI) (*P* = 0.0001), previous congestive heart failure (*P* = 0.003), Charlson co-morbidity index (*P* = 0.03) and Rockwood Frailty Score (*P* = 0.003). Those in the lowest group of baseline serum vitamin D were more likely to have a raised high sensitive C reactive protein hsCRP (high group: 3.50 mg L^−1^ [1.1–7.0] vs. low group: 4.50 mg L^−1^ [1.7–12.0], *P* = 0.042).

At baseline, 34 patients (11.6%) were taking vitamin D supplementation with this increasing to 54 patients (19.4%) at 1 year follow-up. Those in the high serum vitamin D group were more likely to be taking supplementation both at baseline (*P* = 0.011) and at 1 year (*P* = 0.02). 28 patients (10.1%) started supplementation during the follow-up period, 26 patients (9.4%) were on supplementation at both baseline and 1 year with 8 patients (2.9%) stopped supplementation during follow-up.

Overall, 97 patients (33%) within the cohort had prior history of MI and of note the majority (64/97, 66%) of these were in the low serum vitamin D group (low group: n = 64, 43.5% vs. high group: n = 33, 22.4%, *P* < 0.0001). Those in the low group of serum vitamin D had a 2.67 increased odds of presenting in this study with a history of prior myocardial infarction (Odds Ratio [OR] 2.67, 95% confidence interval [CI] 1.61–4.42, *P* < 0.0001). This relationship was slightly attenuated but remained statistically significant when adjusted for any statistically significant baseline differences between groups of serum vitamin D in Table 1 (OR 2.0, 95% CI 1.10–3.60, *P* = 0.02).

There was a higher incidence of congestive cardiac failure (CCF) at baseline in those within the low serum vitamin D group (P = 0.003). Those with a history of CCF had a significantly lower serum vitamin D level at baseline verses those without a history of CCF (16.0 nmol L^-1^ [0–28.0] vs. 31.0 nmol L^-1^ [18.0–54.0], *P* = 0.001), with no difference in vitamin D supplementation (8.8% vs. 8.1%, *P* = 0.89).

There was no difference in non-invasive parameters of cardiovascular status in the high vs. low serum vitamin D group, with similar measures of vascular stiffness (pulse wave velocity 9.21 m s^−1^ (±2.1) vs. 9.42 m s^−1^ (±2.0), *P* = 0.06) and endothelial function (natural log reactive hyperaemia index 0.58 [0.44–0.76] vs. 0.55 [0.44–0.69], *P* = 0.17) respectively.

The composite primary outcome occurred in 76 patients (25.9%) at 1 year. In participants where more than one component of the composite outcome occurred, time-to-first-event was used. There was no difference in the incidence of the composite primary outcome (*P* = 0.12) between groups of serum vitamin D. There was no difference in incidence of any individual component of the primary composite outcome between groups (*P* > 0.05) (**S1 File**). The overall 1 year mortality in this cohort was 5.5%.

**Fig 2:**
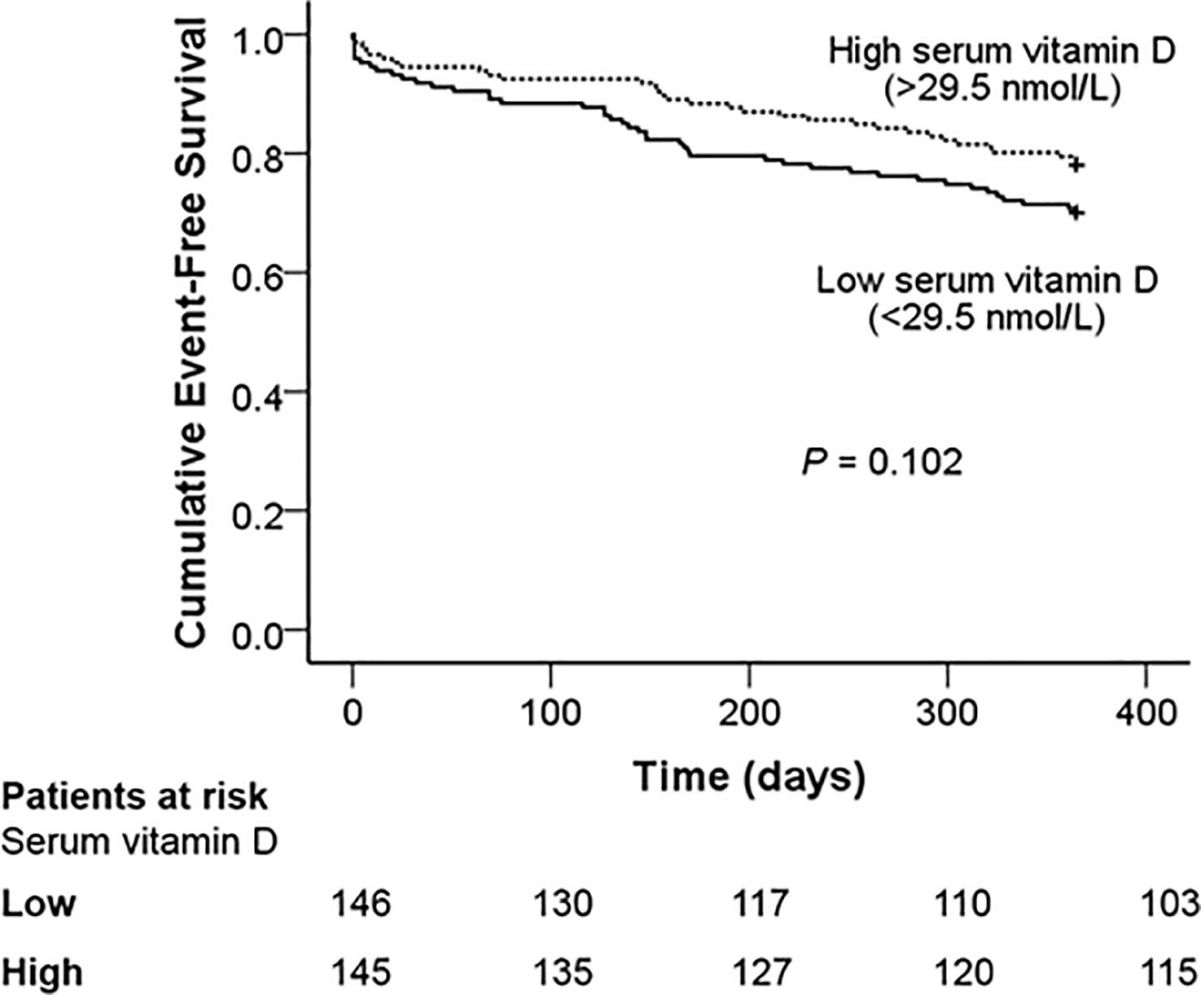
Cumulative event-free survival from incidence of the cumulative primary composite endpoint at 1 year.

In Cox proportional hazard regression models, there was no association between being in the lower group of baseline serum vitamin D and incidence of the primary composite outcome (**Table 2**) in the univariate unadjusted model (Hazard ratio, HR 1.46 [95% confidence interval, CI 0.93–2.30], *P =* 0.10) and the fully adjusted multivariate models (*P* < 0.05). Given the low event rate, these findings were internally validated through bootstrapping of the fully adjusted (Model 4) model, with no difference in hazard ratio and the relationship remaining non significant (HR 1.20 [95% CI 0.72–2.0], *P* = 0.49) (**S1 File**). Kaplan-Meier cumulative event-free survival analysis curves are shown in **Fig 2**, with a non-significant trend for reduced mean survival time in those in the low group of baseline serum vitamin D (*P* = 0.102 by Log-rank test).

**Table 2:** Univariate and multivariate Cox regression for the association between serum vitamin D and the incidence of the composite primary outcome at 1 year.

Incidence of the composite primary outcome^††^ at 1 year	Baseline serum vitamin D	
	Low (<29.5 nmol L^-1^) vs. High† (≥29.5 nmol L^-1^)	
	Hazard ratio (95% confidence interval)	*P value*
**Model 1**	1.46 (0.93–2.30)	0.10
**Model 2**	1.35 (0.84–2.17)	0.21
**Model 3**	1.15 (0.69–1.90)	0.60
**Model 4**	1.20 (0.72–2.0)	0.49

**†** High serum vitamin D used as reference.

**††** Primary outcome is composite of death, myocardial infarction, stroke, unplanned repeat revascularisation or major bleeding.

**Model 1**: Unadjusted analysis.

**Model 2**: Adjusted for age, sex and month of blood collection.

**Model 3**: Additionally adjusted for hypertension, previous myocardial infarction, congestive heart failure, Charlson co-morbidity index, Rockwood Frailty Score, haemoglobin, high sensitivity C-reactive protein.

**Model 4**: Additionally adjusted for vitamin D supplementation at either baseline or 1 year follow up.

The ICON1 vitamin D biomarker sub-study is the largest study to date into the prognostic value of serum vitamin D in high-risk older adults (mean age 80.5 years) undergoing invasive management of NSTEACS. There was no relationship between the baseline serum vitamin D levels and the incidence of the composite primary outcome at 1 year. In this cohort, there was a low prevalence of vitamin D sufficiency, with a median value at baseline (29.5 nmol L^-1^) well below the international cut-off for vitamin D sufficiency (50 nmol L^-1^).

In our study, participants were largely frail, with 68.2% of participants being Rockwood Frailty Score ≥3 or either pre-frail or frail on the Fried Frailty Index. There is an established link between increasing frailty and increased cardiovascular disease risk. In previous analysis of the ICON1 cohort by this group, frailty had a 2.79 increased hazard (95% CI 1.28–6.08, *P* = 0.01) for the primary end-point at 1-year compared to those that were robust.[15] Frailty has been linked to vitamin D deficiency in a number of studies,[24, 25] with our study confirming the finding in older adults presenting with NSTEACS. Those with lower levels of serum vitamin D were found to be significantly more co-morbid, with an increased Charlson co-morbidity index, and be frailer than those with higher levels of serum vitamin D. The relationship between frailty and increased CVD risk, combined with the increased incidence of vitamin D deficiency in older adults, might go some way to explain the excess CVD risk in older adults.

It is well established that low serum vitamin D is associated with an increased incidence of MI,[8, 9] In our study 66% of patients presenting with a history of prior MI at baseline were within the low group of serum vitamin D. Patients with a baseline serum vitamin D <29.5 nmol L^-1^ had twice an adjusted increased odds (95% CI 1.10–3.60, *P* = 0.02) of previously having a MI. In addition, those within the lowest group of serum vitamin D were also more likely to have co-morbid CCF at baseline, with 62.5% of the burden of CCF in the study population being in those in the lowest group. Low serum vitamin D has been shown to be associated with progression of heart failure and may act as an independent predictor of mortality,[26] and is associated with risk of hospitalisation for heart failure.[27] However, vitamin D supplementation has not been shown to prevent incidence of heart failure as primary prevention or significantly influence its clinical course as secondary prevention.[28]

Physiological evidence exists that vitamin D may influence early atherogenesis, potentially through inflammation, and hence be translating into increased CVD incidence in epidemiological studies. High-sensitivity C-reactive protein (hsCRP), a marker of subclinical inflammation has been linked with low levels of serum vitamin D in younger patients.[29, 30] In this current study, those in the low group of serum vitamin D had a statistically significant increase in hsCRP level. This is in keeping with the current literature which suggests vitamin D is anti-inflammatory,[31] modulating both the innate and adaptive immune system,[32] with sufficient levels having an overall reduction in low-grade inflammation characteristic of early atherosclerosis.[33]

At 1 year, there was an increased incidence of the composite primary outcome in those in the low group of serum vitamin D compared to the high group, however this was not significant. Contrary to our findings, some studies have found a significant relationship between vitamin D status at baseline and adverse events at follow-up after ACS, however all are in younger patients, with the inclusion of ST elevation myocardial infarction (STEMI) likely driving up the number of adverse events. Correia et al investigated outcomes after ACS in a sample of 206 adults (mean age 70 years) and concluded those with dichotomised baseline 25-hydroxyvitamin D (25-OH D ≤25 nmol/L had a significantly higher adjusted odds of CVD mortality, which was limited to the early in-hospital period.[13] However verses our study they had a significantly greater number of deaths at 1 year follow-up (24% vs. 5.5%), likely secondary to the recruitment of STEMI (25.2% of study participants) and capture of deaths secondary to complications after coronary artery bypass surgery. Metrio et al investigated 814 patients (mean age 67.0 years) presenting with both NSTEMI (58%) and STEMI (41%) and found those in the lowest quartile of 25-OH D had an increased hazard of mortality.[12] In the largest study to date, Ng et al studied 1259 patients (mean age 65.7 years) with ACS (49.1% STEMI) and found that the lowest quartile of serum 25-OH D significantly predicted adverse outcomes in the fully adjusted model (P = 0.002).[14] In this current study, the serum vitamin D sample was taken during the acute phase of NSTEACS immediately prior to angiography, rather than in a community dwelling cohort. The literature suggests low vitamin D may have effects on the early development of atherosclerotic plaques. However we have shown that in older adults after the NSTEACS event there is no clear association of vitamin levels and future adverse events. Thus vitamin D supplementation might not have any clinical benefit in older patients with NSTEACS.

The role of vitamin D supplementation as primary or secondary prevention is controversial. As would be expected, in our study those on vitamin D supplementation were more likely to have a higher serum vitamin D. However, evidence suggesting any translation into a benefit in clinical outcomes is unclear. Despite the link discussed in observational studies, the interventional evidence is mixed with only a small number of trials specifically designed to assess CVD outcomes.[34] The VITamin D and OmegA-3 TriaL (VITAL) study, a two-by-two factorial randomised control trial, investigated the impact of daily vitamin D_3_ supplementation verses placebo as primary prevention for CVD in 25,871 participants (mean age 67.1 years). The study found no evidence of a protective effect of vitamin D supplementation verses placebo on incidence of a composite of myocardial infarction, stroke or death from cardiovascular causes (HR 0.96, 95% CI 0.85–1.12, *P* = 0.69) over a median follow-up of 5.3 years. However, there is currently no large RCT evidence of vitamin D supplementation as secondary prevention for CVD in older adults. Given the high incidence of vitamin D deficiency in older adults, vitamin D supplementation as secondary prevention to prevent cardiac events requires further study, particularly as the increased risk over younger patients cannot be explained by traditional cardiovascular risk factors alone.

This current study has some limitations. The overall event was low, in particular stroke events, which might be a reflection of the inclusion of selected patient cohort, however the findings were internally validated via bootstrapping and were found to remain non-significant. Only one blood sample was taken and therefore the absolute values should be considered a snap shot of a patient’s biochemical status rather than an average over a longer period. This study is observational and the findings can only be considered hypothesis generating. It would have been better to take into account the racial differences in terms of adverse outcomes. However our population consisted of mostly Caucasian patients and therefore ethnic comparison was not possible. It has been suggested that any beneficial or protective effects of vitamin D therapy will likely be evident only in clinical trials recruiting at least thousands of patients, particularly for hard end points such as cardiovascular mortality[35]. However the recent Vital trial found no evidence of a protective effect of vitamin D supplementation verses placebo on incidence of a composite of myocardial infarction, stroke or death from cardiovascular causes[36].

## Conclusions

In this older cohort of patients presenting with NSTEACS undergoing invasive management, the baseline serum vitamin D level does not predict the incidence of adverse outcomes at 1 year. Our data suggest baseline serum vitamin D level, taken at time of angiography, is not a useful biomarker to predict adverse outcomes after invasive management in this older, high-risk cohort with NSTEACS. Older patients with a prior history of MI are more likely to have a significantly lower serum vitamin D, suggesting further trials investigating vitamin D supplementation for secondary prevention in older adults are needed.

## Supporting information

S1 File Serum 25-hydroxyvitamin D assay platform analysis. Incidence of the composite primary end-point at 1 year. Internal validation using bootstrapping of the association between baseline serum vitamin D and the incidence of the composite primary outcome at 1 year in the fully adjusted (Model 4) multivariate Cox regression model.
(DOCX)
