# Supplementary material for: The association between vitamin D status and clinical events in high-risk older patients with non-ST elevation acute coronary syndrome undergoing invasive management
Source: PLoS One. 2019 Jun 12;14(6):e0217476. doi: 10.1371/journal.pone.0217476 (PMC6561555; doi:10.1371/journal.pone.0217476)
Supplement: S1 File — Incidence of the composite primary end-point at 1 year. Internal validation using bootstrapping of the association between baseline serum vitamin D and the incidence of the composite primary outcome at 1 year in the fully adjusted (Model 4) multivariate Cox regression model. (DOCX) [file pone.0217476.s001.docx]

**Supporting information S1 File**

Serum 25-hydroxyvitamin D assay platform analysis.

| **Time period of recruitment** | **Patients analysed at baseline (n=)** | **Name of assay platform** | **Lower limit of detection (nmol L^-1^)** | **Measurement range**  **(nmol L^-1^)** |
| --- | --- | --- | --- | --- |
| November 2012 – July 2013 | 59 | DiaSorin Liaison | 10 | 10 - 374 |
| August 2013 – May 2015 | 191 | Roche Diagnostics Modular E170 | 10 | 10 - 250 |
| June 2015 – December 2015 | 44 | Roche Cobas 602 | 12.5 | 12.5 - 150 |

Incidence of the composite primary end-point at 1 year.

|  | **Overall** | **Baseline serum vitamin D** | | |
| --- | --- | --- | --- | --- |
|  |  | **High**  ≥29.5 nmol L^-1^ | **Low**  <29.5 nmol L^-1^ | *P*  *value* |
| **Composite primary outcome at 1 year**, n (%) | 76 (25.9) | 32 (21.9) | 44 (29.9) | 0.12 |
| Death, n (%) | 16 (5.5) | 4 (2.7) | 12 (8.2) | 0.07^†^ |
| Myocardial infarction, n (%) | 30 (10.2) | 14 (9.6) | 16 (10.9) | 0.72 |
| Stroke, n (%) | 4 (1.4) | 2 (1.4) | 2 (1.4) | 1.0^†^ |
| Unplanned repeat revascularisation, n (%) | 21 (7.2) | 11 (7.5) | 10 (6.8) | 0.81 |
| Bleeding, n (%) | 32 (10.9) | 14 (43.8) | 18 (12.2) | 0.47 |

^†^ Fisher exact test

Internal validation using bootstrapping of the association between baseline serum vitamin D and the incidence of the composite primary outcome at 1 year in the fully adjusted (Model 4) multivariate Cox regression model.

|  | **Unbootstrapped** | | **Bootstrapped for 1000 samples** | |
| --- | --- | --- | --- | --- |
|  | HR  (95% CI) | P value | HR  (95% CI) | 95% CI |
| **Baseline serum vitamin D** |  |  |  |  |
| **High**^†^  ≥29.5 nmol L^-1^ | Reference | Reference | Reference | Reference |
| **Low**  <29.5 nmol L^-1^ | 1.20  (0.72 – 2.0) | 0.48 | 1.20  (0.72 – 2.0) | 0.48 |
